# Obesity-Related Disorders in Türkiye: A Multi Center, Retrospective, Cross-Sectional Analysis from the OBREDI-TR Study

**DOI:** 10.3390/jcm14082680

**Published:** 2025-04-14

**Authors:** Alihan Oral, Ihsan Solmaz, Nizameddin Koca, Ulas Serkan Topaloglu, Ismail Demir, Ahmet Dundar, Ali Kirik, Ozge Kama Basci, Hacer Sen, Emine Binnetoglu, Nalan Okuroglu, Ahmet Aydin, Zeynep Irmak Kaya, Hamit Yildiz, Aycan Acet, Gokhan Tazegul, Hasan Sozel, Osman Ozudogru, Kubilay Issever, Selcuk Yaylacı, Ugur Bayram Korkmaz, Nur Duzen Oflas, Celalettin Küçük, Kamil Konur, Teslime Ayaz, Aysun Isiklar, Esref Arac, Hilmi Erdem Sumbul, Huseyin Ali Ozturk, Ali Burak Govez, Yusuf Usame Durmus, Atilla Onmez, Sibel Ocak Serin, Nazif Yalcin, Aysegul Ertinmaz, Alper Tuna Guven, Mehmet Kok, Yasin Sahinturk, Seyit Uyar

**Affiliations:** 1 Department of Internal Medicine, Faculty of Medicine, Biruni University, Halkalı Street No. 99, 34295 İstanbul, Türkiye; dr.alihanoral@gmail.com; 2 Department of Internal Medicine, Diyarbakir Gazi Yasargil Education Research Hospital, 21070 Diyarbakir, Türkiye; ihsan2157@gmail.com; 3 Department of Internal Medicine, Health Sciences University Bursa Health Application and Research Center, Bursa City Hospital, 16250 Bursa, Türkiye; nkoca@yahoo.com (N.K.); nazifyalcin16@gmail.com (N.Y.); aertinmaz@yahoo.com (A.E.); 4 Department of Internal Medicine, Kayseri City Hospital, 38080 Kayseri, Türkiye; ustop38@gmail.com; 5 Department of Internal Medicine, Bozyaka Education Research Hospital, 35170 Izmir, Türkiye; drismaildemir22@gmail.com; 6 Department of Internal Medicine, Mardin Savur Prof. Dr. Aziz Sancar State Hospital, 47860 Savur, Türkiye; drahmetdundar@hotmail.com; 7 Department of Internal Medicine, Faculty of Medicine, Balikesir University, Altieylül, 10145 Balikesir, Türkiye; alikirik87@hotmail.com (A.K.); ozgee.kama@gmail.com (O.K.B.); hcrgrsy@hotmail.com (H.S.); 8 Department of Internal Medicine, Corlu Vatan Hospital, 59860 Corlu, Türkiye; edemirbas1@yahoo.com; 9 Department of Internal Medicine, Fatih Sultan Mehmet Education Research Hospital, 34752 Istanbul, Türkiye; nokuroglu@yahoo.com; 10 Department of Internal Medicine, Faculty of Medicine, Medipol University, Bagcilar, 34214 Istanbul, Türkiye; uzm.dr.ahmetaydin@gmail.com; 11 Department of Internal Medicine, Health Sciences University Eskisehir Health Application and Research Center, Eskisehir City Hospital, 26080 Eskisehir, Türkiye; dr.zeynepirmak@gmail.com; 12 Department of Internal Medicine, Faculty of Medicine, Gaziantep University, 27600 Sehitkamil, Türkiye; drhyildiz@haotmail.com; 13 Department of Internal Medicine, Faculty of Medicine, Kutahya Health Sciences University, 43020 Kutahya, Türkiye; aycanacet80@icloud.com; 14 Department of Internal Medicine, Faculty of Medicine, Marmara University, 34854 Istanbul, Türkiye; drgtazegul@gamil.com; 15 Department of Internal Medicine, Faculty of Medicine, Akdeniz University, 07100 Antalya, Türkiye; hasansozel@akdeniz.edu.tr; 16 Department of Internal Medicine, Faculty of Medicine, Erzincan Binali Yildirim University, 24100 Erzincan, Türkiye; osmanozudogru2@gmail.com; 17 Department of Internal Medicine, Giresun University Education Research Hospital, 28100 Giresun, Türkiye; kubilayissever@gmail.com; 18 Department of Internal Medicine, Faculty of Medicine, Sakarya University, 54100 Sakarya, Türkiye; selcukyaylaci@sakarya.edu.tr; 19 Department of Internal Medicine, Izmir Katip Celebi Education Research Hospital, 35360 Izmir, Türkiye; ugurbk07@gmail.com; 20 Department of Internal Medicine, Faculty of Medicine, Van Yuzuncu Yil University, 54100 Van, Türkiye; dr.nurdzn@hotmail.com; 21 Vocational School, Biruni University, 34295 İstanbul, Türkiye; 22 Department of Internal Medicine, Faculty of Medicine, Recep Tayyip Erdogan University, 53020 Rize, Türkiye; 23 Department of Internal Medicine, Bakircay University Cigli Education Research Hospital, 36610 Izmir, Türkiye; drteslimeayaz@gmail.com; 24 Department of Internal Medicine, Acibadem Atasehir Hospital, Atasehir, 34642 Istanbul, Türkiye; aysunisiklar@gmail.com; 25 Department of Internal Medicine, Faculty of Medicine, Dicle University, 21010 Diyarbakir, Türkiye; esrefarac@gmail.com; 26 Department of Internal Medicine, Health Sciences University Adana Health Application and Research Center, Adana City Hospital, 01230 Adana, Türkiye; esumbul@cu.edu.tr (H.E.S.); drozturkhuseyinali@gmail.com (H.A.O.); abgovez@gmail.com (A.B.G.); drmusyusuf80@gmail.com (Y.U.D.); 27 Department of Internal Medicine, Faculty of Medicine, Duzce University, 81000 Duzce, Türkiye; attilaonmez@gmail.com; 28 Department of Internal Medicine, Umraniye Education Research Hospital, Umraniye, 34764 Istanbul, Türkiye; rdsibelocak@gmail.com; 29 Department of Internal Medicine, Faculty of Medicine, Baskent University, 06790 Ankara, Türkiye; alper.tuna.guven@gmail.com; 30 Department of Internal Medicine, Antalya Education Research Hospital, 07080 Antalya, Türkiye; dr.mehmetkok@hotmail.com (M.K.); drsahinturk@yahoo.com (Y.S.); seyituyar79@hotmail.com (S.U.)

**Keywords:** obesity, chronic diseases, prevalence

## Abstract

**Objectives**: Obesity is a significant public health concern, as it is associated with the development of numerous chronic diseases. The prevalence of obesity and attendant diseases has been increasing over recent years. This study attempted to ascertain the frequency of chronic diseases in obese patients in Türkiye for the first time on this scale. **Methods**: A retrospective study was conducted, with patients admitted to the internal medicine outpatient clinics or obesity centers between December 2023 and December 2024 included in this study. Participants were recruited from seven regions, 20 provinces, and 28 centers, and the inclusion criteria were met by those aged 18 years and over with a body mass index (BMI) of 30 kg per square meter (kg/m^2^) or above. Their status, with respect to chronic diseases, and their anthropometric parameters were documented. **Results**: The total number of patients was 10,121, with a mean age of 45.2 ± 13.92. Of these, 7222 (71.35%) were female. The prevalence of type 2 diabetes mellitus (T2DM), hypertension (HT), dyslipidemia (DL), coronary artery disease (CAD), obstructive pulmonary disease (OPD), obstructive sleep apnea syndrome (OSAS), and fatty liver disease (FLD) was found to be 35.01%, 78.19%, 12.37%, 10.32%, 5.88%, and 75.12%, respectively. A subsequent analysis of the prevalence of these diseases by region revealed a statistically significant variation between regions (*p* < 0.001 for all regions). **Conclusions**: This study represents a substantial contribution to the existing body of knowledge in this field, particularly with regard to the identification of the current chronic disease rate of obese patients in Türkiye.

## 1. Introduction

Obesity is a multifaceted condition that can lead to numerous chronic complications and is associated with an increased risk of mortality. It develops due to the interaction of genetic, environmental, and behavioral factors [1,2]. According to the Global Burden of Disease Report by the World Health Organization (WHO), one in eight people worldwide was affected by obesity in 2022. The report reveals that approximately 2.5 billion adults were overweight and 890 million were obese; since 1990, there has been a more than twofold increase in global adult obesity, and a fourfold increase in adolescent obesity [3]. The issue of obesity has been identified as having significant ramifications for both health and the global economy. According to the WHO, the financial implications of obesity are set to be a significant concern in the coming years. By 2035, the organization has estimated that the global economic impact of obesity will reach an estimated 4 trillion dollars [4]. Notably, in recent years, the increase in obesity prevalence in high-income countries has attracted particular attention. However, there has been an increase in the prevalence of obesity in low- and middle-income countries in recent years. Unhealthy eating habits and low levels of physical activity are leading to an increase in obesity, particularly in countries where urbanization is increasing rapidly [3]. As reported by the WHO, 60% of adults within the European Region are classified as overweight or obese; in countries such as the United Kingdom, Germany, and Italy, there has been a rapid increase in obesity rates over the last decade. Türkiye has the distinction of having the highest percentage of overweight or obese citizens among European Countries, with 66% of the population falling into these categories [5].

A study in Türkiye revealed that sensory appeal, convenience, and mood were the three most significant factors influencing food choice across different regions. Conversely, weight control was found to be the least important factor. These variations may be attributed to cultural differences between the regions [6]. A significant contributing factor to the prevalence of overweight and obesity is the lack of physical activity. A study conducted by the Ministry of Health of the Republic of Türkiye examined the physical activity habits of 15,468 adults in seven provinces selected from seven regions, revealing that only 3.5% of the participants engaged in regular physical activity [7].

Obesity is defined as an excess of adipose tissue in the body. Subcutaneous adipose tissue accumulation is the normal physiological buffer for excess energy intake. Once the storage capacity of this tissue is exceeded, fat accumulates in other areas, forming visceral adipose tissue (VAT) [8]. Chronic inflammation caused especially by VAT, VAT-induced increased levels of free fatty acids and leptin synthesis, and decreased adiponectin levels are the leading causes of insulin resistance and atherosclerosis [9]. Chronic diseases such as type 2 diabetes mellitus (T2DM), cardiovascular diseases, dyslipidemia, and hypertension occur on this basis in obese individuals [9]. Specifically, it has been demonstrated that mesenteric and retroperitoneal visceral adipose tissue is strongly associated with obesity-related diseases, including cardiovascular diseases and T2DM in obese patients [8]. Obesity also has been demonstrated to be a contributing factor to several serious health complications, including certain types of cancer and respiratory disorders [10]. In a study conducted in 2019, it was estimated that deaths resulting from non-communicable diseases (NCDs) such as heart diseases, T2DM, malignant neoplasms, cerebrovascular diseases, chronic pulmonary diseases, and gastrointestinal diseases, among others, amounted to an estimated 5 million deaths. The aforementioned diseases are associated with a body mass index (BMI) higher than the optimal range [11]. According to recent estimates, the prevalence of overweight and obesity has been identified as the fourth most common modifiable risk factor for NCDs in the European region, surpassed only by hypertension, dietary risks, and tobacco [5].

According to the International Diabetes Federation’s (IDF) Diabetes Atlas 2021, the prevalence of DM has increased to 10.5% in adults aged 20–79 years; the WHO states that approximately 1.3 billion people worldwide live with hypertension; and the proportion of patients with low-density lipoprotein (LDL) above target values is around 75% worldwide [12,13]. Current data further estimates that approximately 350 million people worldwide have coronary artery disease (CAD) [12]. Among adults aged between thirty and seventy years, the prevalence of obstructive sleep apnea syndrome (OSAS) ranges from 24% to 26% in men and from 17% to 28% in women [14].

The treatment of obesity necessitates a multifaceted approach, encompassing not only a reduction in weight but a holistic perspective that addresses the metabolic, cardiovascular, and psychological complications associated with obesity. It is widely acknowledged that the most effective strategy for controlling obesity in the general population is to prevent the onset of unhealthy weight gain. This is because it is generally considered challenging to reverse excess weight gain once it has occurred [15].

The objective of this study was to ascertain the prevalence of obesity-related diseases, including T2DM, hypertension (HT), dyslipidemia, CAD, fatty liver disease (FLD), obstructive pulmonary disease (OPD), and OSAS, within the Turkish population, and to assist in developing strategies for managing these diseases.

## 2. Material and Methods

### 2.1. Patient Selection

Patients admitted to internal medicine outpatient clinics or obesity centers between 15 December 2023 and 31 December 2024 were included in the present study. Patients were recruited from 7 regions, 20 provinces, and 28 centers across the country (Marmara, Southeastern Anatolia, Central Anatolia, Mediterranean, Aegean, Black Sea, and Eastern Anatolia). The inclusion criteria encompassed participants aged 18 and above with a BMI of 30 kg per square meter (kg/m^2^) or higher. Exclusion criteria included a history of any type of cancer, type 1 DM, and obesity caused by known genetic or endocrine disorders.

### 2.2. Data Collection

At admission, patients underwent a standard evaluation that measured height, weight, waist circumference, blood pressure, and data collection on smoking status, age, and sex. Patients were categorized into three distinct groups, contingent upon their smoking status: namely, never smokers, former smokers, and current active smokers. Waist circumference (WC) was evaluated in the horizontal plane, with measurement conducted at the midpoint between the lowest rib and the anterior superior iliac spine. The evaluation of the patient’s chronic diseases was based on the current laboratory findings and drug utilization information from the preceding three months, as recorded in the national health system database and the patient’s statements about existing diseases. The presence of chronic diseases, including T2DM, hypertension, dyslipidemia, CAD, obstructive pulmonary diseases, OSAS, and FLD, was determined and recorded. Diagnoses of these chronic diseases were made according to Table 1. Imaging modalities were preferred over metabolic parameters in diagnosing fatty liver disease. Patients with any of the current ultrasonography (USG), elastography, computed tomography (CT), magnetic resonance imaging (MRI) data, and fatty deposits detected on these imaging modalities were diagnosed with a fatty liver. This study was conducted following the guidelines of the Declaration of Helsinki and approved by the Ethics Committee of Biruni University (2024/84 and 19 November 2024).

**Table 1:** Diagnostic Criteria.

Diabetes mellitus [16]	HBa1c of greater than or equal to 6.5% Fasting glucose greater than or equal to 126 mg/dL Random blood glucose of greater than or equal to 200 mg/dL with any symptom related to diabetes Oral Glucose Tolerance Test: Two-hour blood glucose of greater than or equal to 200 mg/dL or use of antidiabetic drugs with a previously confirmed diagnosis of T2DM based on the above criteria
Hypertension [17]	As defined by ESH 2024, equal or above 140/90 mmHg arterial blood pressure or use of anti-hypertensive drugs previously with a previously confirmed diagnosis of HT based on the above criteria
Dyslipidemia [18]	LDL over 70 mg/dL and/or HDL under 50 mg/dL and/or total cholesterol over 200 mg/dL and/or triglycerides above 150 mg/dL or taking an anti-hyperlipidemic drug. (Sensitivity analysis performed using LDL ≥ 130 mg/dL for comparison in Table 2).
Coronary artery disease [19]	Previous MI, coronary bypass surgery history, stent, or stenosis confirmed by coronary angiography
Obstructive pulmonary disorders [20]	Asthma or COPD confirmed by RFT
Obstructive sleep apnea syndrome [21]	Confirmed diagnosis with polysomnography
Fatty Liver Disease [22]	Fatty deposits determined by non-invasive imaging (USG/Elastography/CT/MRI)

Hba1c (glycated hemoglobin), LDL (low-density lipoprotein), HDL (high-density lipoprotein), mg/dl (milligrams per decilitre), ESH (European Society of Hypertension), mmHg (millimeter of mercury), MI (myocardial infarction), COPD (chronic obstructive pulmonary disease), RFT (respiratory function test), USG (ultrasonography), CT (computerized tomography), MRI (magnetic resonance imaging). Dyslipidemia is primarily defined as LDL ≥ 70 mg/dL as recommended by cardiovascular guidelines. For sensitivity analysis, LDL ≥ 130 mg/dL was also considered, with the prevalence shown in Table 2.

**Table 2:** Regional distribution of patients with chronic diseases.

	Aegean (n = 1139)	Marmara (n = 3436)	Central Anatolia (n = 1314)	Black Sea (n = 797)	Eastern Anatolia (n = 452)	Southeastern Anatolia (n = 1824)	Mediterranean (n = 1159)	FDR-Adjusted *p*-Value
DM	509(44.7)	1272(37.0)	294(22.4)	316(39.6)	154(34.1)	535(29.3)	399(34.4)	<0.001
HT	714(62.7)	1852(53.9)	596(45.4)	512(64.2)	188(41.6)	879(48.2)	744(64.2)	<0.001
DL70	1088(95.5)	2502(72.8)	1002(76.3)	591(74.2)	416(92.0)	1410(77.3)	882(76.1)	<0.001
DL130	392(34.4)	657(19.1)	371(28.2)	232(29.1)	134(29.6)	440(24.1)	258(22.3)	<0.001
OPD	186(16.3)	233(6.8)	168(12.8)	55(6.9)	42(9.3)	181(9.9)	89(6.8)	<0.001
CAD	152(13.3)	408(11.9)	107(8.1)	81(10.2)	83(18.4)	218(12.0)	116(11.9)	<0.001
OSAS	72(6.3)	126(3.7)	68(5.2)	15(1.9)	10(2.2)	161(8.8)	50(3.7)	<0.001
FLD	640(56.2)	1802(52.4)	336(25.6)	266(33.4)	176(38.9)	514(28.2)	459(39.6)	<0.001

Data were presented as numbers and (percentages). DM (diabetes mellitus), HT (hypertension), DL70 (dyslipidemia for a cut-off of 70 mg/dL), DL130 (dyslipidemia for a cut-off of 130 mg/dL), OPD (obstructive pulmonary disease), CAD (coronary artery disease), OSAS (obstructive sleep apnea syndrome), FLD (fatty liver disease).

### 2.3. Statistics

All statistical analyses were performed using SPSS version 26 (Chicago, IL, USA). Continuous variables were presented as a mean ± standard deviation (SD), and categorical variables were presented as counts and percentages. The normality of distribution was assessed using the Kolmogorov–Smirnov test.

To explore the independent associations between demographic and behavioral factors and the presence of chronic comorbidities, multivariate logistic regression analyses were conducted for each of the following outcomes: DM, HT, dyslipidemia, CAD, OPD, OSAS, and FLD. Each model included the following covariates: age (continuous), sex (binary), BMI (continuous), smoking status (categorized as a non-smoker, ex-smoker, or current smoker), and geographic region (seven-category variable, with Marmara as the reference).

For hepatic steatosis, a subset analysis was conducted on participants with available ultrasound data (n = 5581). Collinearity among independent variables was tested using the variance inflation factor (VIF), and all variables were found to be within acceptable limits (VIF < 5). Odds ratios (ORs), 95% confidence intervals (CIs), and *p*-values were reported for each predictor variable. A two-tailed *p*-value < 0.05 was considered statistically significant.

The false discovery rate (FDR) correction was applied using the Benjamini–Hochberg procedure for the chi-square tests to control the inflation of the Type I error rate due to multiple comparisons across regions. A threshold of q < 0.05 was used to determine statistical significance after correction.

To assess the association between age and comorbidity prevalence, participants were categorized into four age groups: <30, 30–44, 45–59, and ≥60 years. Chi-square tests were applied to compare the prevalence of each comorbidity across these age groups. A *p*-value < 0.05 was considered statistically significant.

## 3. Results

In total, 10,121 patient records from 20 cities and 28 centers across seven regions were collated for this study, with the distribution of these records according to region as follows: 3436 from the Marmara region, 1824 from the Southeastern Anatolia region, 1314 from the Central Anatolia region, 1159 from the Mediterranean region, 1139 from the Aegean region, 797 from the Black Sea region, and 452 from the Eastern Anatolia region.

The patients’ mean age was 45.2 ± 13.92 years, with a minimum (min) of 18 and a maximum (max) of 81 years of age. The total number of females was 7.222 (71.35%). The patients’ mean height was found to be 163.90 ± 9.41 cm, with a mean weight of 100.15 ± 18.08 kg, and a mean body mass index of 37.24 ± 6.04 kg/m^2^; the min–max values were 140–198, 69–253, and 30.00–83.60, respectively. The mean systolic blood pressure recorded was 134.05 ± 27.45 mm of mercury (mmHg), and the mean diastolic blood pressure was 80.88 ± 8.23 mmHg; the min–max values were 90–200 and 65–133, respectively. The prevalence for smoking was recorded as follows: active smoking 30.77%, current smoking 11.65%, and never smoking 57.56% (Table 3).

**Figure 1:**
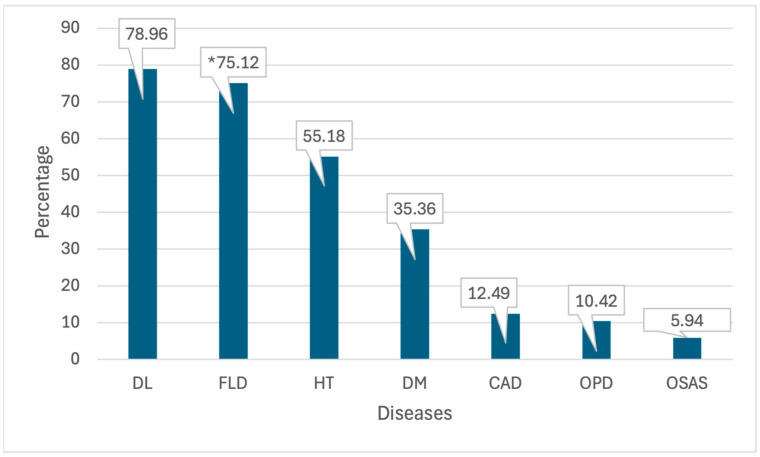
Chronic disease frequency of patients. DM (diabetes mellitus), HT (hypertension), DL (dyslipidemia), OPD (obstructive pulmonary disease), CAD (coronary artery disease), OSAS (obstructive sleep apnea syndrome), FLD (fatty liver disease). * A total of 5581 patients with imaging data for fatty liver disease were evaluated. The percentage calculation is thus based on 5581 patients.

T2DM was diagnosed at 35.01%, hypertension at 54.64%, dyslipidemia at 78.19%, coronary artery disease at 12.37%, obstructive pulmonary disease at 10.32%, OSAS at 5.88%, and FLD at 75.12%. (Figure 1)

A notable finding is the predominance of specific diseases within specific regions. For instance, the Aegean region has the highest prevalence of T2DM, dyslipidemia, hyperlipidemia, obstructive pulmonary diseases, and hepatosteatosis, while the Mediterranean region has the highest prevalence of hypertension. The southeastern Anatolia region has the highest prevalence of OSAS, and Eastern Anatolia has the highest prevalence of cardiovascular diseases (Table 2).

Regarding dyslipidemia, Table 2 presents the prevalence estimates based on two different LDL-C thresholds: ≥70 mg/dL (DL70) and ≥130 mg/dL (DL130). Notably, the choice of threshold significantly affects the observed distribution, with DL70 yielding markedly higher prevalence rates across all regions. This comparison highlights the selected cut-off’s methodological impact on regional disease burden patterns.

### Multivariate Analysis of Risk Factors for Comorbidities

Multivariate logistic regression analyses were performed to determine the independent predictors of each comorbidity among obese individuals. The models included age, sex, BMI, smoking status (non-smoker, ex-smoker, smoker), and geographic region (Marmara as reference).

For DM, age (OR: 1.013, 95% CI: 1.010–1.016, *p* < 0.001) and ex-smoker status (OR: 1.38, 95% CI: 1.21–1.58, *p* < 0.001) were significant predictors, while individuals residing in Central Anatolia and Southeastern Anatolia had lower odds of DM.

Hypertension (HT) was strongly associated with age (OR: 1.088, 95% CI: 1.080–1.088, *p* < 0.001) and ex-smoker status (OR: 1.50, 95% CI: 1.29–1.74, *p* < 0.001).

Dyslipidemia was independently associated with both current smoking (OR: 1.73, 95% CI: 1.54–1.94, *p* < 0.001) and ex-smoking (OR: 1.40, 95% CI: 1.19–1.65, *p* < 0.001), with the highest regional risk observed in the Aegean (OR: 7.70) and Eastern Anatolia (OR: 4.61).

CAD was significantly predicted by ex-smokers (OR: 2.38) and current smoker status (OR: 1.45), while residence in Central Anatolia was associated with decreased odds.

COPD and OSAS were both strongly associated with smoking history and age, while FLD (available in n = 5581) was predicted by age and current smoking.

All models were tested for multicollinearity, and variance inflation factors (VIFs) were below acceptable thresholds (VIF < 5). The results are summarized in Table 4 and illustrated in Figure 1.

**Figure 2:**
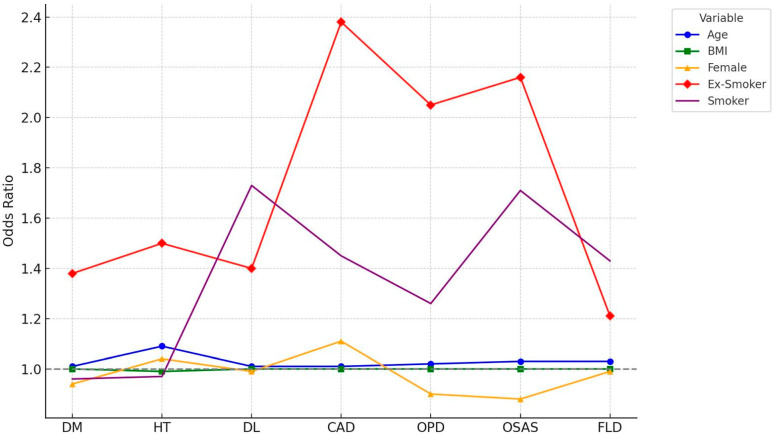
Comparison of Risk Factors Across Comorbidities.

Figure 2 illustrates the adjusted odds ratios (ORs) for five key predictors—age, BMI, female sex, ex-smoker status, and current smoker status—across seven obesity-related comorbidities: diabetes mellitus (DM), hypertension (HT), dyslipidemia (DL), coronary artery disease (CAD), obstructive pulmonary disease (OPD), obstructive sleep apnea syndrome (OSAS), and fatty liver disease (FLD). The grey dashed line indicates the null effect (OR = 1).

**Figure 3:**
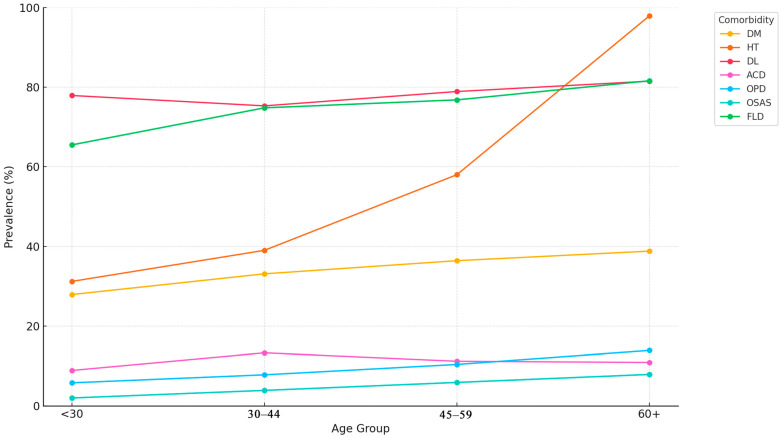
Prevalence of Comorbidities by Age Group.

The prevalence of comorbidities varied significantly across the age groups. For example, the prevalence of hypertension increased from 31.2% in participants aged <30 years to 97.9% in those aged ≥60 years. Similar age-related trends were observed for diabetes mellitus, dyslipidemia, and fatty liver disease. Chi-square tests confirmed that all observed differences in prevalence across the age groups were statistically significant (*p* < 0.001 for all comparisons) (Table 5, Figure 3).

Figure 3 presents a line graph illustrating the prevalence (%) of obesity-related comorbidities across the four age groups: <30 years, 30–44 years, 45–59 years, and ≥60 years. Comorbidities include diabetes mellitus (DM), hypertension (HT), dyslipidemia (DL), coronary artery disease (CAD), obstructive pulmonary disease (OPD), obstructive sleep apnea syndrome (OSAS), and fatty liver disease (FLD). A marked increase in prevalence is observed with advancing age, particularly for HT, DM, and FLD. Percentages are derived from cross-sectional descriptive analysis. Chi-square tests confirmed statistical significance across the age groups.

## 4. Discussion

This multicenter study is among the most comprehensive investigations on obesity-related comorbidities in Türkiye. By encompassing diverse geographical regions and a large sample size, it provides a valuable snapshot of the disease burden associated with obesity, reinforcing the need for early identification and intervention strategies.

Obesity is a key driver of chronic conditions, such as hypertension, diabetes, dyslipidemia, fatty liver disease, and cardiovascular disorders. Our findings align with the existing evidence that links increased adiposity to elevated risks for these diseases [1,23,24,25] Notably, 55.8% of the obese patients in our sample had hypertension, and 35.4% had type 2 diabetes—figures consistent with, or slightly higher than, previous national estimates [26,27,28,29,30,31].

We also observed a high prevalence of dyslipidemia (78.96%) based on LDL ≥ 70 mg/dL. Given our study’s cross-sectional design and screening nature, a lower LDL threshold was selected to increase sensitivity [18]. However, recognizing the variability in definitions across studies, we conducted a sensitivity analysis using the conventional threshold of LDL ≥ 130 mg/dL, which yielded a much lower prevalence of 37.7%. This highlights the importance of threshold selection in epidemiological comparisons, and supports the need for context-specific criteria [18,32].

Additionally, age and ex-smoker status emerged as the most consistent independent predictors across comorbidities. These findings emphasize the long-term metabolic burden of smoking, even after cessation, and the cumulative effect of aging on chronic disease risk [33,34]. Interestingly, BMI did not consistently predict comorbidities, possibly reflecting the heterogeneity within obese populations, as previously described in the context of metabolically healthy vs. unhealthy obesity phenotypes [35].

Our regional analyses revealed significant geographic disparities in comorbidity prevalence. Unmeasured cultural, dietary, and socioeconomic factors may influence these differences. Tailoring public health strategies to regional contexts may improve their effectiveness in addressing obesity-related conditions [36].

The age-stratified analysis further demonstrated a transparent gradient in disease burden, with the prevalence of nearly all comorbidities increasing with age. This finding supports early prevention efforts, particularly among younger individuals who may appear metabolically healthy despite carrying excess weight [29,37].

While our study is strengthened by its size and national coverage, it has limitations. Its retrospective nature and reliance on electronic medical records may introduce misclassification bias. Additionally, although BMI is a practical tool for assessing obesity in large populations, it lacks sensitivity to variations in fat distribution and body composition. The reliance on imaging for diagnosing FLD also introduces a selection bias, as imaging was not uniformly applied to all patients [38,39].

Our findings underscore the complex interplay between obesity and chronic disease and highlight the need for early, targeted, and region-specific interventions to mitigate its growing burden.

## 5. Conclusions

Obesity is not a single disease entity but rather a constellation of interconnected conditions that significantly elevate the risk of chronic diseases. This study demonstrates that obesity-related comorbidities vary substantially across the age groups and geographic regions in Türkiye, underscoring the need for tailored public health strategies. The identification of obesity should prompt simultaneous screening for related conditions, such as hypertension, diabetes, dyslipidemia, and hepatic steatosis, particularly in high-risk subgroups, such as older adults and ex-smokers.

Given the lack of prior nationwide studies examining the chronic disease burden among obese individuals in Türkiye, this work fills a significant gap and provides a data-driven foundation for action. We recommend the development of region-specific screening protocols, lifestyle modification programs, and risk communication campaigns targeting clinicians and the public. These measures are essential to reduce preventable morbidity and mortality, and to address the growing burden of obesity in a targeted, population-sensitive manner.

## Figures and Tables

**Table 3:** Baseline Characteristics of Patients.

Age (year)	45.20 ± 13.92
Female sex n (%)	7222
Height (cm)	163.90 ± 9.41
Weight (kg)	100.15 ± 18.08
Body mass index (BMI) (kg/m^2^)	37.24 ± 6.04
Systolic BP (mmHg)	134.17 ± 27.45
Diastolic BP(mmHg)	80.88 ± 8.23
Smoking status	
Smoker n (%)	3146(31.08)
Ex-smoker n (%)	1191(11.76)
Non-smoker n (%)	5884(58.13)

Data were presented as a mean ± standard deviation or number and percentage. BP (blood pressure).

**Table 4:** Multivariate Logistic Regression Results for Comorbidities.

Comorbidity		Age	BMI	Female	Ex-Smoker	Smoker
DM	OR	1.01	1.00	0.94	1.38	0.96
	CI (95%)	(1.01–1.02)	(0.99–1.00)	(0.86–1.03)	(1.21–1.58)	(0.87–1.05)
	*p*-value	*p* = 0.000	*p* = 0.295	*p* = 0.218	*p* = 0.000	*p* = 0.372
HT	OR	1.09	0.99	1.04	1.50	0.97
	CI (95%)	(1.08–1.09)	(0.99–1.00)	(0.94–1.15)	(1.29–1.74)	(0.88–1.07)
	*p*-value	*p* = 0.000	*p* = 0.131	*p* = 0.434	*p* = 0.000	*p* = 0.564
DL	OR	1.01	1.00	0.99	1.40	1.73
	CI (95%)	(1.00–1.01)	(0.99–1.01)	(0.89–1.11)	(1.19–1.65)	(1.55–1.94)
	*p*-value	*p* = 0.000	*p* = 0.636	*p* = 0.911	*p* = 0.000	*p* = 0.000
CAD	OR	1.01	1.00	1.11	2.38	1.45
	CI (95%)	(1.00–1.01)	(0.99–1.01)	(0.96–1.28)	(1.99–2.83)	(1.26–1.67)
	*p*-value	*p* = 0.030	*p* = 0.495	*p* = 0.145	*p* = 0.000	*p* = 0.000
OPD	OR	1.02	1.00	0.90	2.05	1.26
	CI (95%)	(1.02–1.03)	(0.99–1.01)	(0.77–1.04)	(1.69–2.49)	(1.08–1.47)
	*p*-value	*p* = 0.000	*p* = 0.755	*p* = 0.166	*p* = 0.000	*p* = 0.003
OSAS	OR	1.03	1.00	0.88	2.16	1.71
	CI (95%)	(1.02–1.04)	(0.98–1.02)	(0.72–1.07)	(1.66–2.81)	(1.40–2.10)
	*p*-value	*p* = 0.000	*p* = 0.984	*p* = 0.205	*p* = 0.000	*p* = 0.000
FLD	OR	1.03	1.00	0.99	1.21	1.43
	CI (95%)	(1.02–1.03)	(0.99–1.01)	(0.86–1.14)	(0.99–1.49)	(1.24–1.66)
	*p*-value	*p* = 0.000	*p* = 0.797	*p* = 0.911	*p* = 0.067	*p* = 0.000

OR (odds ratio), CI (confidence interval), DM (diabetes mellitus), HT (hypertension), DL (dyslipidemia), OPD (obstructive pulmonary disease), CAD (coronary artery disease), OSAS (obstructive sleep apnea syndrome), FLD (fatty liver disease).

**Table 5:** Prevalence of Comorbidities by Age Group (%).

Comorbidity	<30	30–44	45–59	60+	*p*-Value
DM	27.9%	33.1%	36.4%	38.8%	<0.001
HT	31.2%	39.0%	57.0%	97.8%	<0.001
DL	77.9%	75.3%	78.9%	81.5%	<0.001
CAD	8.9%	13.3%	11.2%	10.9%	<0.001
OPD	5.8%	7.8%	10.4%	13.9%	<0.001
OSAS	2.0%	3.9%	5.8%	7.9%	<0.001
FLD	65.5%	74.8%	76.8%	81.6%	<0.001

DM (diabetes mellitus), HT (hypertension), DL (dyslipidemia), OPD (obstructive pulmonary disease), CAD (coronary artery disease), OSAS (obstructive sleep apnea syndrome), FLD (fatty liver disease).

## Data Availability

The datasets used and analyzed during the current study are available from the corresponding author upon reasonable request.
